# Interocular conflict from a monocular augmented reality display: Impact of visual characteristics on performance

**DOI:** 10.1371/journal.pone.0256766

**Published:** 2021-09-02

**Authors:** Elodie Bayle, Sylvain Hourlier, Sylvie Lelandais, Charles-Antoine Salasc, Laure Leroy, Justin Plantier, Pascaline Neveu

**Affiliations:** 1 Thales AVS, France SAS, Mérignac, France; 2 French Armed Forces Biomedical Research Institute, Brétigny-sur-Orge, France; 3 IBISC, Evry University, Paris Saclay University, Evry, France; 4 Paris 8 University, Saint-Denis, France; Justus Liebig Universitat Giessen, GERMANY

## Abstract

In monocular see-through augmented reality systems, each eye is stimulated differently by a monocular image that is superimposed on the binocular background. This can impair binocular fusion, due to interocular conflict. As a function of visual characteristics, the latter can have a greater or lesser impact on user comfort and performance. This study tested several visual characteristics of a binocular background and a monocular element during an exposure that reproduced the interocular conflict induced by a monocular see-through near-eye display. The aim was to identify which factors impact the user’s performance. Performance was measured as target tracking and event detection, identification, fixation time, and latency. Our results demonstrate that performance is a function of the binocular background. Furthermore, exogenous attentional stimulation, in the form of a pulse with different levels of contrast applied to the monocular display, appears to preserve performance in most background conditions.

## Introduction

Vision refers to how a living being uses information from the visual system to meet its needs. In humans, the visual system integrates information from two sensors, the eyes. When these two pieces of information are compatible, binocular fusion ensures that the brain establishes a single (rather than dual) perception of the scene. Binocular fusion is composed of motor fusion, which aligns the visual axes of each eye on the fixed object, and sensory fusion, where these two images are combined to form one. The latter is triggered by images from each of the two retinas, and these images must be sufficiently similar for it to occur [1]. Motor fusion, on the other hand, can only be accurate in the case of sensorially mergeable targets [2].

When using a monocular see-through near-eye display, a binocular background is, by definition, common to both eyes and can be fused, while the virtual image (called symbology in the aeronautical context) is only superimposed on one eye. Consequently, the images that are presented to the two eyes are not perfectly compatible, creating interocular conflict. In the aeronautical context, pilots who use a monocular see-through near-eye display, such as a helmet mounted display (HMD), report degraded perception of visual cues, static and dynamic visual illusions, and visual discomfort [3, 4]. Hale and Piccione [5] argue that some of the asthenopia experienced by users of monocular see-through near-eye display is due to binocular rivalry, although no visual suppression was reported in their study. However, in monocular augmented reality, interocular conflict is specific, as the difference between the two images is limited to a small area, while the rest of the visual field can be merged. A few studies have established that perception through a monocular see-through near-eye display is a function of the visual characteristics of the environment. The following results have been observed in previous studies:

- The predominance (*i*.*e*. percentage of time during which a stimulus is dominant) of the monocular stimulus over the binocular background is greater when [6]:
  ○ the monocular image has high contrast (21.9 compared to 4.6);
  ○ the monocular image has high luminance (8 fL = 27.4 cd/m^2^ compared to 0.28 fL = 0.96 cd/m^2^);
  ○ background luminance is low (0.28 fL = 0.96 cd/m^2^ compared to 8 fL = 27.4 cd/m^2^);
  ○ the HMD has good resolution (630 compared to 165 TVL);
  ○ the field of view of the HMD is narrow (15° compared to 45° viewing angle);
  ○ the background complexity is low (compared to high);
  ○ the focal plane of the HMD is distant (infinite compared to 30 inches).
- Tracking performance is not significantly different between two HMD configurations (“good HMD” in which the monocular image was projected at a resolution of 650 TV and 10 fL versus “poor HMD” in which the monocular image was projected at a resolution of 240 TVL and 0.3 fL) [6].
- The detection time of the events is shorter when they are projected on the binocular background than when they are projected on the monocular image [7, 8].

Hershberger and Guerin [6] asked participants to focus on a static monocular image in an equally static background. However, in a typical monocular augmented reality flying task, mobile monocular see-through HMD symbology is combined with a dynamic binocular background (*e*.*g*. the flight path vector with the runway) while task interruptions are introduced when information appears. Building on the latter study, our aim is to verify whether the reported results remain robust when a dynamic binocular background is used. Furthermore, the latter study limited the assessment of the impact of the interocular conflict to the predominance of the monocular element. Thus, neither background visibility nor performances in a perceptual task were assessed. The latter authors also found that the impact of disturbances was reduced when monocular information was displayed to the dominant eye.

However, more recently, the display eye has not been found to influence perception [7, 9]. Furthermore, Browne et al. [7] (only) evaluated detection performance against a daylight background in clear weather, similar to optimal flight conditions. However, as Hershberger and Guerin [6] suggest, it is likely that changes in environmental conditions that affect the spatial frequency or contrast of the background alter the visibility of the monocular image. Therefore, the impact of the display eye needs to be clarified and evaluated under different display conditions.

In a similar vein, another study evaluated perception when a monocular stimulus was added to two binocular images [10]. The authors report that stimulus characteristics have an impact on perception, as they found that the greater the difference in contrast between the monocular stimulus and the binocular image, the greater the alternation between them.

Although interocular conflict induced by a monocular see-through near-eye display differs from binocular rivalry observed with completely dissimilar images (called dichoptic images) presented to the corresponding regions of the two eyes [11], the literature in this area is extensive and supports an effect of the visual characteristics of the environment on perception during interocular conflict. In this specific context, it has been shown that perception can take two forms. First, the image captured by one eye can be totally suppressed [11]. This leads to an alternation of perception in which one image is perceived exclusively, while the other is suppressed, or vice versa. Before this alternation, which does not appear until a threshold of 150 ms is reached [12], the two images are superimposed (a phenomenon called abnormal fusion). Secondly, a mixed image, composed of patches of the left and right image simultaneously, resembling a mosaic, can also be perceived [13]. Generally, small stimuli (under 2°) generate more exclusive perception than larger stimuli, which tend to generate more mixed rivalry perception [13–16]. Studies that compare pairs of stimuli show that those with higher contrast lead to a higher rate of alternation than those with lower contrast [17, 18]. There is, therefore, less exclusive perception of one of the two stimuli. O’Shea et al. [19] evaluated the interaction of the spatial frequency of dichoptic stimuli on binocular rivalry. They reported that the higher the spatial frequency of the two stimuli, the more mixed perception they generated. The latter study also confirmed that the more extensive the dichoptic stimuli, the more mixed perceptions they generated. Other work has found that when two images differ in terms of contrast, the one with higher contrast dominates, and the rate of alternation increases relative to the condition where the contrast of the two images is the same [20, 21]. Furthermore, images with a higher level of luminance have been found to predominate over those with lower luminance [22, 23], and a moving pattern predominates over a static pattern. The literature has also studied the impact of attention on suppression in binocular rivalry conditions with two dichoptic images. There are two forms of attention. *Endogenous attention* refers to attention that is directed and controlled by the individual. The person voluntarily tries to maintain his or her perception of one stimulus rather than another. It has been shown that, for dichoptic stimuli, endogenous attention has an effect on the initial selection of a stimulus in the presence of binocular rivalry [24]. *Exogenous attention*, on the other hand, is characterized by the presence of external cues that automatically activate attention processes. Ooi and He [25] showed that, in the case of dichoptic stimuli, a focus on a suppressed stimulus stops the suppression, and makes it dominant. Chong and Blake [24] confirmed this effect, but only for stimuli displayed for more than 400 ms.

Paffen and Van der Stigchel [26] observed that deleting an image increased the perceived contrast between images, thus increasing the probability of alternation at the moment of the deletion. Involuntary attention can, they argue, initiate perceptual alternation. This observation seems to be consistent with a recent study by Naber et al. [27], which shows that when dichoptic stimuli are displayed, an abrupt change in the contrast of one of them solicits exogenous attention, leading to more mixed perception and, therefore, less suppression.

In monocular see-through near-eye display context, Winterbottom et al. [28] found that endogenous attention does not improve target recognition detection time. However, the latter authors did not evaluate the effect of exogenous attention on detection time or performance, measured as target recognition. Therefore, the question arises as to its impact on interocular conflict in this context.

The present study extends earlier research, and assesses the impact of interocular conflict on performance and visual comfort under different display conditions. Our aim was to identify the impact of visual characteristics on performance and comfort. Specifically, we asked participants to align a monocular moving element with a dynamic binocular background. Performance was measured by tracking the movement of a binocular element over a binocular background with a monocular element. During the task, monocular or binocular events were randomly displayed at different eccentricities, and participants were asked to detect and identify them. Performance was also measured by detection time and the accuracy of event identification. An eye tracker was used to measure latency between the moment the stimulus appeared and the triggering of the eye movement towards it. Event fixation time was measured and several visual characteristics were tested–in particular, spatial frequencies and the contrast of the binocular background. As the impact of contrast, attention, and display eye, in the context of monocular see-through near-eye display remains unclear, we assessed their influence by modulating these parameters with respect to the moving monocular element. Performance criteria were, thus, evaluated at different spatial frequencies and contrast levels with respect to the binocular background, and the characteristics of the monocular element.

Binocular rivalry can cause symptoms such as visual fatigue, headaches and visual suppression [29]. Browne and Moffit [30] found, using a scale adapted from Babbitt and Nystrom [31], that a monocular stimulus superimposed on a binocular background generated more visual discomfort than a stimulus projected on a screen. Other, more general studies of fatigue among pilots wearing a see-through HMD report complaints of visual fatigue and eye disorders related to monocular see-through HMD [3–5, 9]. We therefore also used a measure of visual comfort to evaluate if the interocular conflict generated by our stimulus resulted in symptoms.

Partial or total suppression of an image can be caused by binocular rivalry. Motor fusion can be altered by this suppression, and by the fact that each eye sees two different images that are difficult to merge. We therefore ran three optometric tests to determine if participants’ motor fusion was impacted by interocular conflict.

In sum, our study evaluates three hypotheses, notably that: the visual characteristics of the image (the binocular background and the monocular element); exogenous attention; and display eye impact performance in a context of interocular conflict akin to monocular see-through near-eye display.

## Materials and methods

### Ethics statement

The study was conducted in accordance with the Helsinki Declaration and was reviewed and approved by the *Comité de protection des personnes EST* I, a French research ethics committee (N IDRCB: 2018-A01331-54). Volunteers provided an informed consent prior to the experiment, and were informed of their right to end their participation at any time.

### Participants

Eighteen participants were enrolled in the study: twelve women and six men aged 21–45 years (M = 32; SD = 7.3). Inclusion criteria were: uncompensated ametropia limited to ±1.00 D, measured with the Nidek AR600A (Nidek, Aichi, Japan) autorefractometer; compensated far (6 m) and near vision (0.4 m) acuity of ≤ 0.03 log units for each eye; and anisometropia of less than 2.00 D. The limit of stereoscopic acuity was set at ≤ 120 arcseconds. The participant was required to be aware of the phenomenon of physiological diplopia, and have no motor, neurological or oculomotor problems.

### Apparatus

Monocular see-through near-eye displays such as see-through HMD generate interocular conflict because two different images are seen by both eyes. One eye perceives only the background while the other perceives both the background and the augmented image.

Using a projector-based system, it is possible to reproduce this conflict via active glasses combine with stereoscopic projectors in which each eye can also have a dedicated image. Since the aim of our study is only to assess the impact of visual characteristics on performance, the participant’s head was kept fixed so that we did not have to deal with the slaving of the scene to their head.

**Fig 1:**
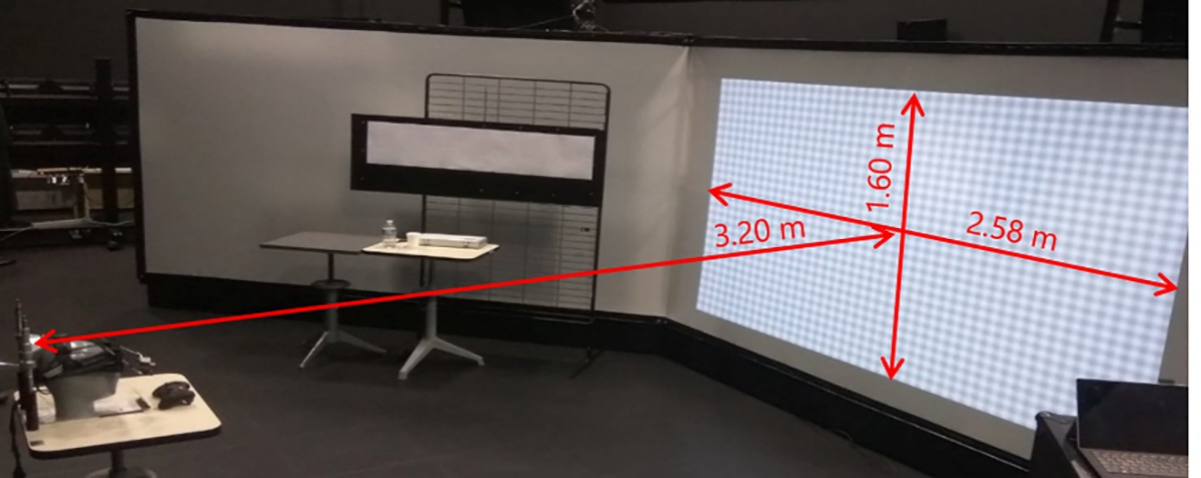
The experimental setup. The participant was seated 3.20m from a screen 1.6m high and 2.58m wide. The HSF 100% condition is captured in this figure.

The participant was seated 3.20 m from a screen (1.6 m high, 2.58 m wide) upon which two images were projected at 120 Hz using a stereoscopic video projector (F80-4k12, Barco, Courtrai, Belgium) (see Fig 1).

He or she wore active occlusion glasses (Edge RF, Volfoni®, Paris, France), which displayed a dedicated image, at 60 Hz, to each of the two eyes. The monocular image was projected on the screen with the rest of the image but so that only one eye could see it. This display reproduces the interocular conflict generated by monocular see-through near-eye displays in which a monocular image is superimposed on a binocular background.

**Fig 2:**
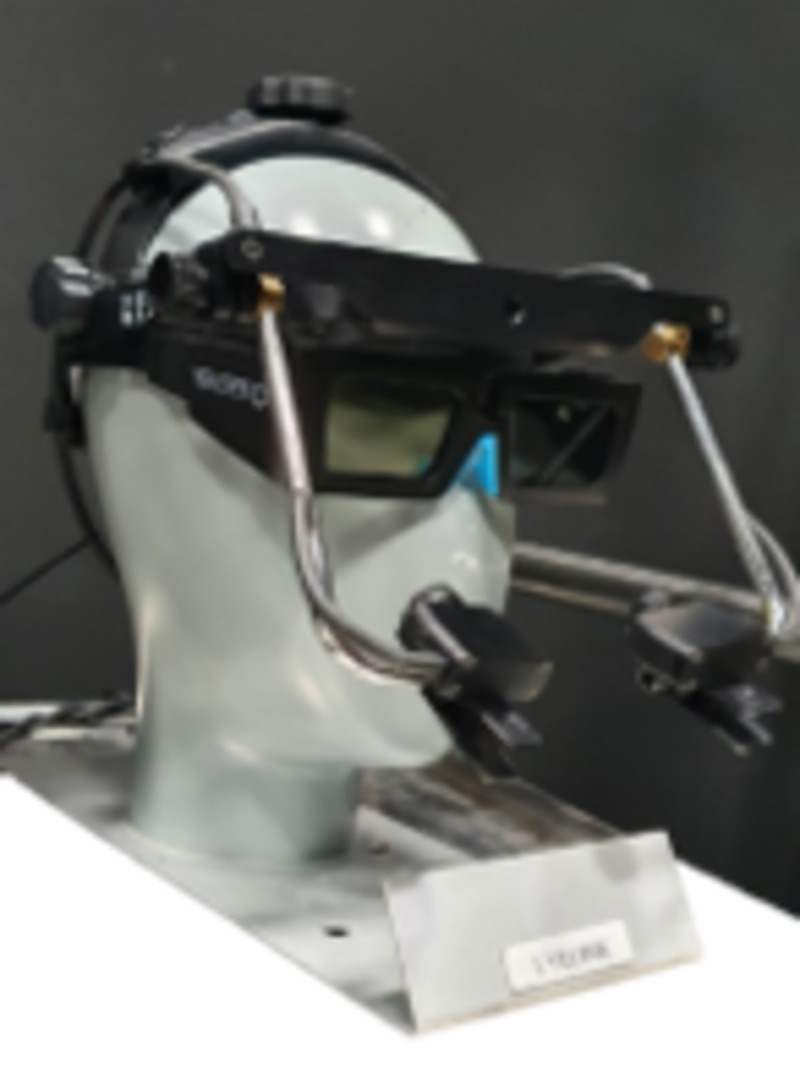
Eye tracker and occlusion glasses worn by the participant.

The participant also wore an eye tracker (EyeLink II, Eyelink®, Ottawa, Canada) that measured gaze position over time, and his or her head was fixed via a chinrest to ensure that it remained in the same position during each condition (see Fig 2).

**Fig 3:**
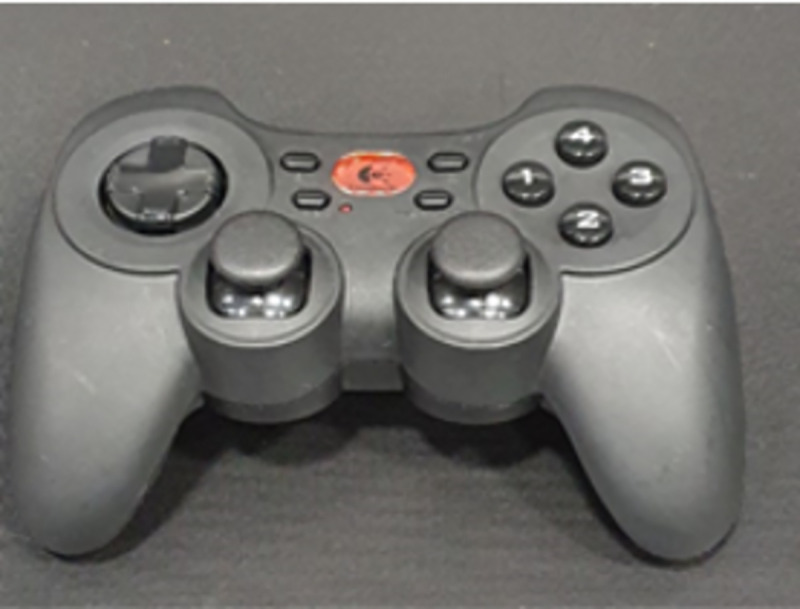
Two-handed game controller used to carry out tasks.

A game controller (Cordless Rumblepad 2, Logitech®, Lausanne, Switzerland) was used to perform the tasks (see Fig 3).

To evaluate the effect of stimuli on motor fusion, we used a modified Thorington scale with a Maddox cylinder, and a pen torch to measure phoria. Fusional reserves were also measured, which required the use of a horizontal prism bar of 1 to 45 D. Forced vergence was evaluated using a Wesson card, polarized glasses and three prisms of 3 Δ, 6 Δ and 10 Δ.

Visual comfort was assessed using a questionnaire. Participants were asked to give an oral score, ranging from 0 to 5 (0 being no symptoms, 5 being an intolerable symptom), in relation to the stimulation generated by the condition with respect to his or her feeling of: visual fatigue; headaches; double vision; blurred vision; eye strain; difficulty concentrating; and nausea and dizziness.

### Stimuli

Stimuli were generated using the MATLAB Psychophysics Toolbox (The MathWork, Natick, MA, USA).

Mean screen luminance was 13 cd/m^2^.

**Fig 4:**
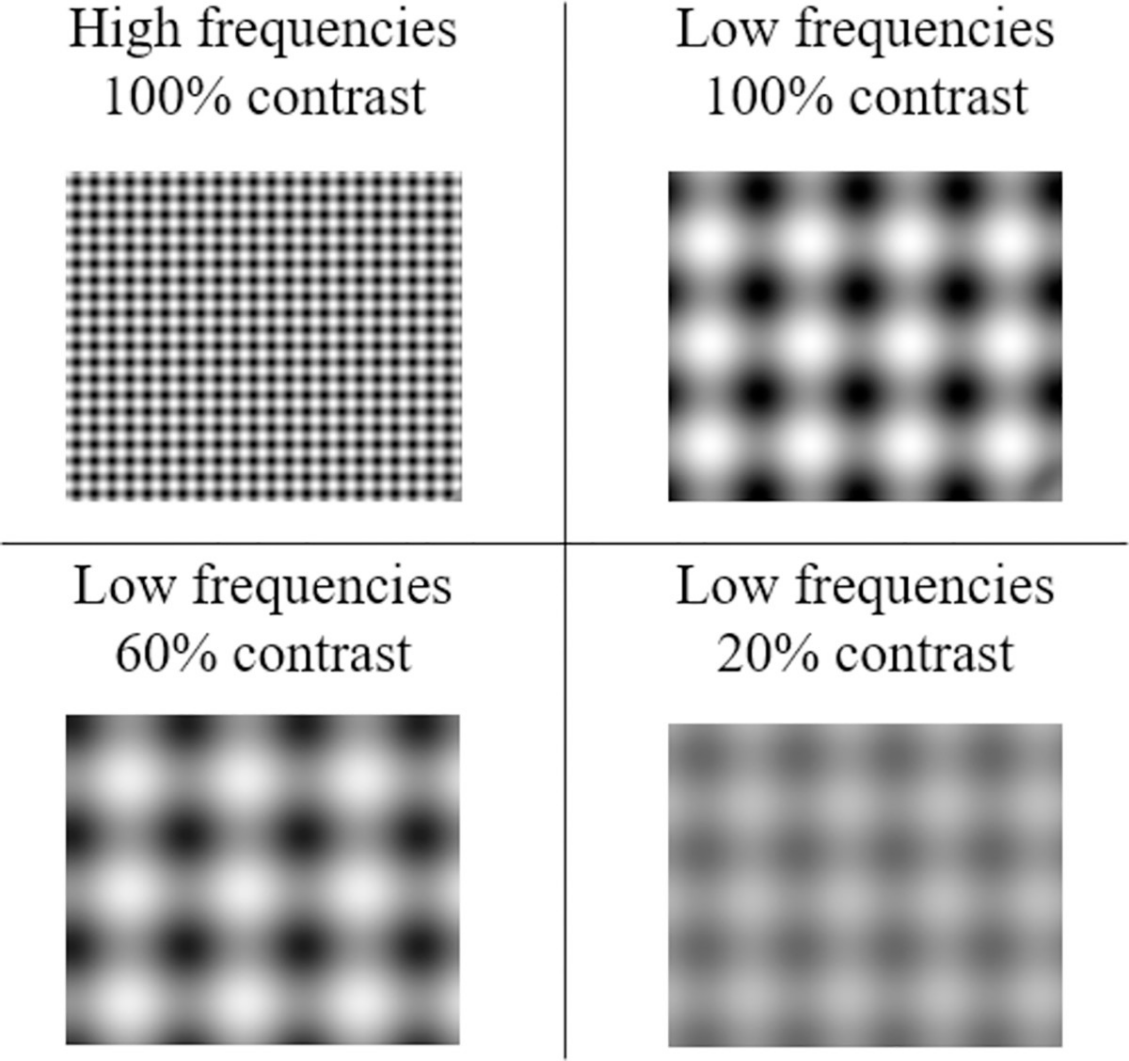
Binocular background according to the four display conditions: HSF-100%, LSF-100%, LSF-60% and LSF-20%.

In order to identify the impact of spatial frequency and contrast, the binocular background could be displayed at two (high and low) spatial frequencies (6.92 cpd and 1 cpd), and three levels of contrast: 100%, 60%, and 20% of the maximum screen luminance (26 cd/m^2^) (see Fig 4).

**Fig 5:**
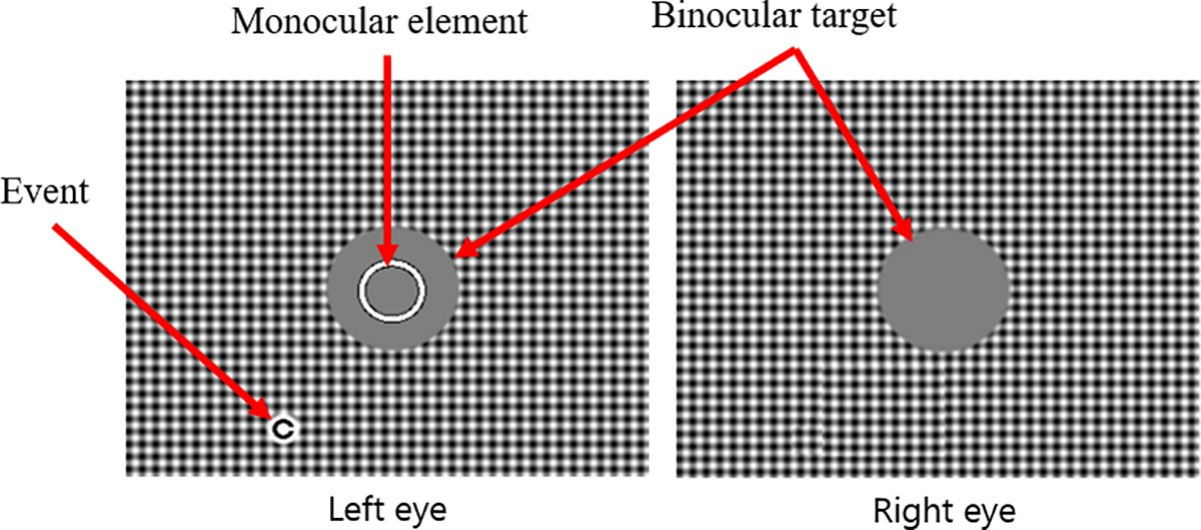
Stimuli displayed to the participant’s left and right eyes via the stereoscopic equipment. Images can be merged by the participant. The condition shown is a combination of a binocular background at 100% contrast and 6.92 cpd (HSF-100%), during the display of a monocular Landolt ring at 2° eccentricity on the left eye.

The background was free of semantic content and context, and clearly specified with respect to spatial frequency and contrast. It was composed of vertical and horizontal sinusoidal gratings, with a circular target (2.1°) that followed the movement of the background (see Fig 5). The purpose of the sinusoidal gratings was to facilitate motor fusion of the background. The binocular background and the binocular circular target moved at a speed of 2°/s on an identical trajectory for all conditions.

**Fig 6:**
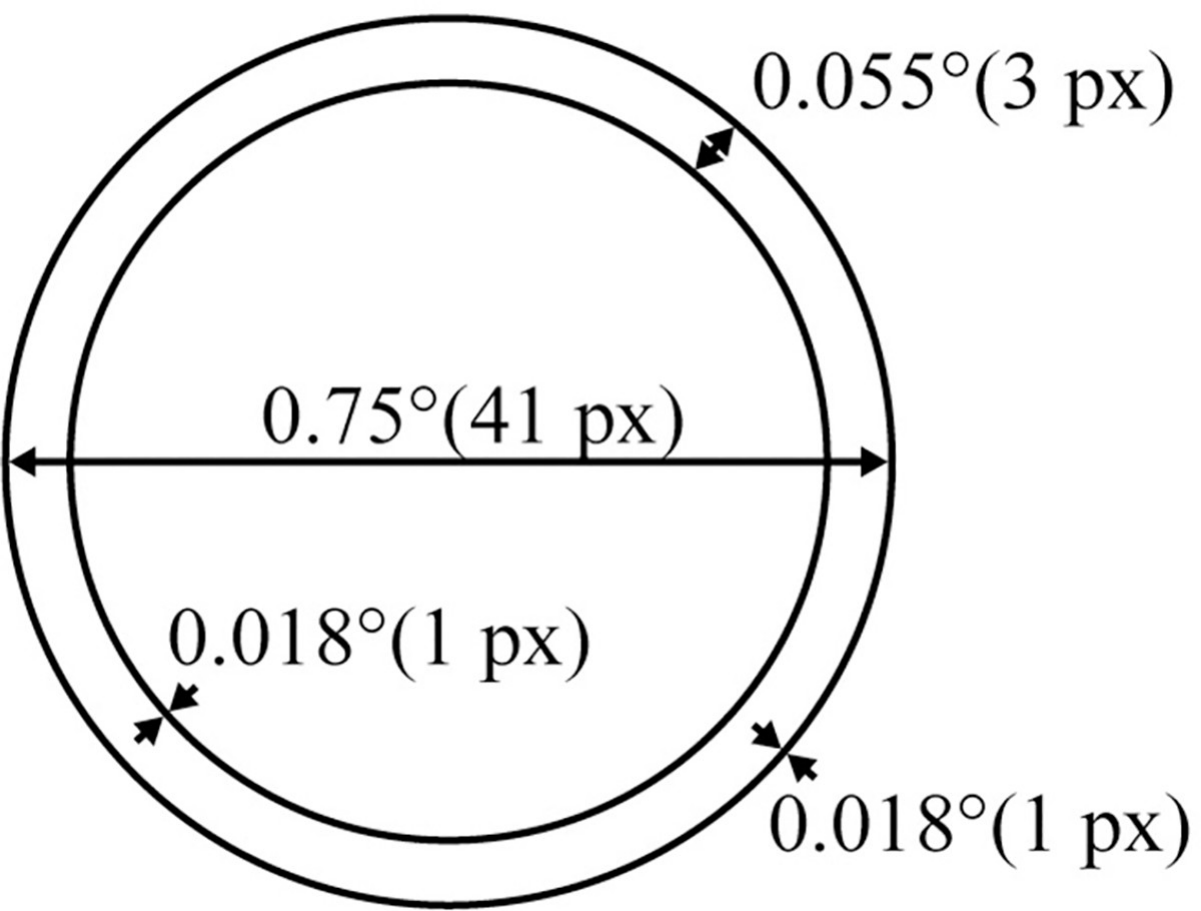
Monocular element in the form of a ring.

Participants were asked to align a monocular element (see Fig 6) with the circular binocular moving target. The monocular element took the form of a 0.75° ring.

The events to be identified were Landolt rings randomly displayed between 500 and 1500 ms every 5000 to 8000 ms binocularly or monocularly. All rings were displayed with the same size (0.48°) and with 100% contrast; the goal was to ensure that their identification was only dependent on the display (binocular vs monocular). They were presented in four directions of aperture (i.e., opening to the left, right, up, or down), at two eccentricities (0.81° and 2.00° visual angle) randomly around the center of the target. Sixteen events per condition had to be identified. The monocular element, the target and the events were all projected and rendered on the background plane.

**Fig 7:**
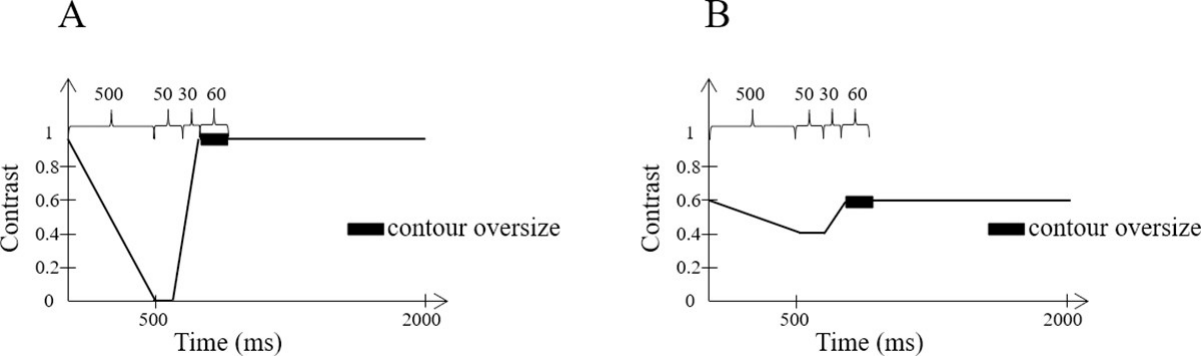
Contrast variation of the monocular element over a two-second period for the high contrast condition (A) and the intermediate contrast condition (B).

Our aim was to determine the impact of contrast, attention, and the display eye in the context of monocular see-through near-eye display. The monocular element contrast was either set to 1 (high contrast) or 0.6 (intermediate contrast). Exogenous attention was evaluated by modulating the contrast of the monocular element from 0 to 1 for high contrast, and from 0.4 (10.4 cd/m^2^) to 0.6 for intermediate contrast. This contrast modulation is referred to as *pulsing*. The pulsing profile varied as a function of the contrast condition of the monocular element (see Fig 7). The contrast of the latter was set to decrease for 500 ms, then remain constant for 50 ms, then increase for 30 ms. Its thickness was then doubled for 60 ms, before recovering its original size, until 2000 ms had passed.

In practice, as for dichoptic stimuli where the stimulus with the highest contrast dominates [17, 18, 27], pulsing can predictably modify the contrast between the background and the monocular element. The effect is to equalize predominance and, consequently, avoid detrimental information loss. It was therefore necessary to check whether this pattern improved target tracking without degrading event detection and identification.

**Fig 8:**
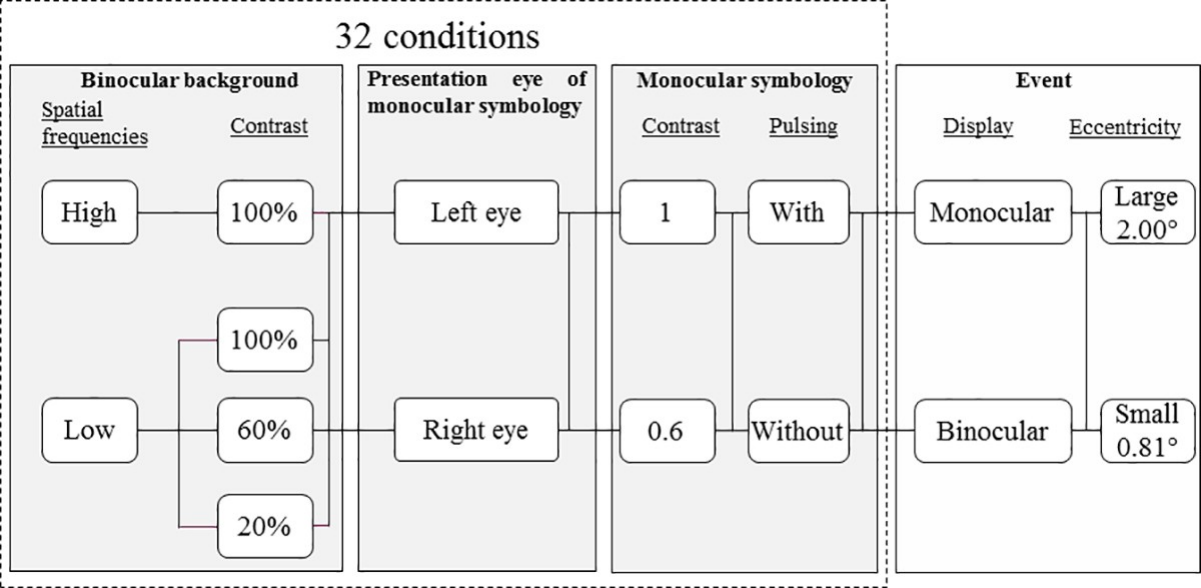
The 32 tested conditions. The independent variables that constitute these 32 conditions comprise binocular background and monocular element characteristics and display eye. In each condition, Landolt rings characteristics were also modified.

A total of 32 conditions were tested (see Fig 8), as follows:

- 4 background conditions: one with a background at high spatial frequency and 100% contrast (HSF-100%) and 3 with a background at low spatial frequency and 100, 60 or 20% contrast (LSF-100%, LSF-60%, LSF-20%);
- x 2 eyes (the monocular element was randomly placed on the right or the left eye);
- x 2 maximum contrasts for the monocular element (1 or 0.6);
- x 2 pulsing conditions (with or without).

### Tasks

The tracking task consisted of making the center of the dynamic monocular element, which could have a maximum speed of at 4°/s, coincide with the center of the binocular moving target (2°/s), using the game controller joystick with the right thumb.

At the same time, the participant was asked to detect and identify an event. He or she had to indicate, as quickly as possible, the direction of a Landolt ring opening (which could be up, down, right or left), using the arrow keys on the game controller with the left thumb. The participant was able to correct any mistakes before the next event appeared, and an error was only recorded when their last response and the orientation of the ring were different. If the participant lost sight of part (potentially a mixed perception) or all of the background or the monocular element, he or she could report this by pressing one of the four game controller triggers.

### Procedure

First, the participant was provided with written instructions, asked to give written consent and inclusion criteria were checked.

Given the large number of conditions and the fact that each condition required calibration and a long image loading time, each participant’s test was divided into two sessions.

The 32 conditions were balanced between the two sessions to avoid any training effect. Of the eight repetitions of each background condition (eight for the HSF-100%, eight for the LSF-100%, eight LSF60% and eight LSF20%), four were performed in the first session and four in the second. Of the sixteen conditions performed with the right eye, eight were performed in the first session, eight in the second and so on for each independent variable. Once these allocation rules were followed, the conditions were randomized.

Each condition lasted about 2 minutes and each session lasted 2 hours and 30 minutes.

The three optometric tests were run in each session. They were ordered from the most dissociative to the most associative in order to preserve, as far as possible, potential effects on motor fusion. The first test consisted in measuring the participant’s phoria at 3 m using a modified *Thorington* method. The second test measured their fusion amplitude at 3 m, and the third measured fixation disparity and forced vergence at 0.40 m.

Next, the participant was seated in front of the screen. He or she was equipped with the eye tracker and active glasses, and his or her chin was supported on a chin rest to maintain the head in a stable position for the duration of each condition. The eye tracker was calibrated before each condition using thirteen points remained fixed. An initial familiarization test was run to ensure that the participant had understood the instructions and was comfortable using the game controller. 16 conditions were conducted one after the other. At the end of this first session, the optometric tests were repeated, in the same order as before.

There was a delay of at least one day between the first session and the second session. The second session followed the same procedure as the first: optometric tests were performed, followed by a familiarization run, then the other 16 conditions, and finally the three optometric tests were repeated.

### Data processing

Data were analyzed using MATLAB R2019a with customized scripts. The assessment of motor fusion was based on data collected in the three optometric tests (see Table 3).

Performance was evaluated using the following variables:

- *target tracking*;
- *quality of event identification*;
- *event detection time*;
- *latency* of the oculomotor response to the appearance of an event;
- *event fixation time*;
- *loss of information*.

*Target tracking* performance was measured as the error (in arc minutes) between the center of the monocular element and the center of the binocular target. This measure was averaged for each condition. *Quality of event identification* performance was evaluated by calculating the number of correct event identifications for each condition. *Event detection* performance was assessed as the detection time (in milliseconds) between the appearance of the event and the response of the participant using the game controller. The *latency of the oculomotor response* to the appearance of an event was measured via the eye tracker, by evaluating the latency between the appearance of an event and the initiation of the eye movement towards it, corresponding to the first speed peak after at least five consecutive points exceeded 2°/sec [32]. *Event fixation time* was recorded for each event as a function of the display condition. This was determined as the time interval (in milliseconds) between the first eye movement (corresponding to the first speed peak of at least 2°/second) and the return to the monocular element (the second speed peak, opposite to the first).

**Fig 9:**
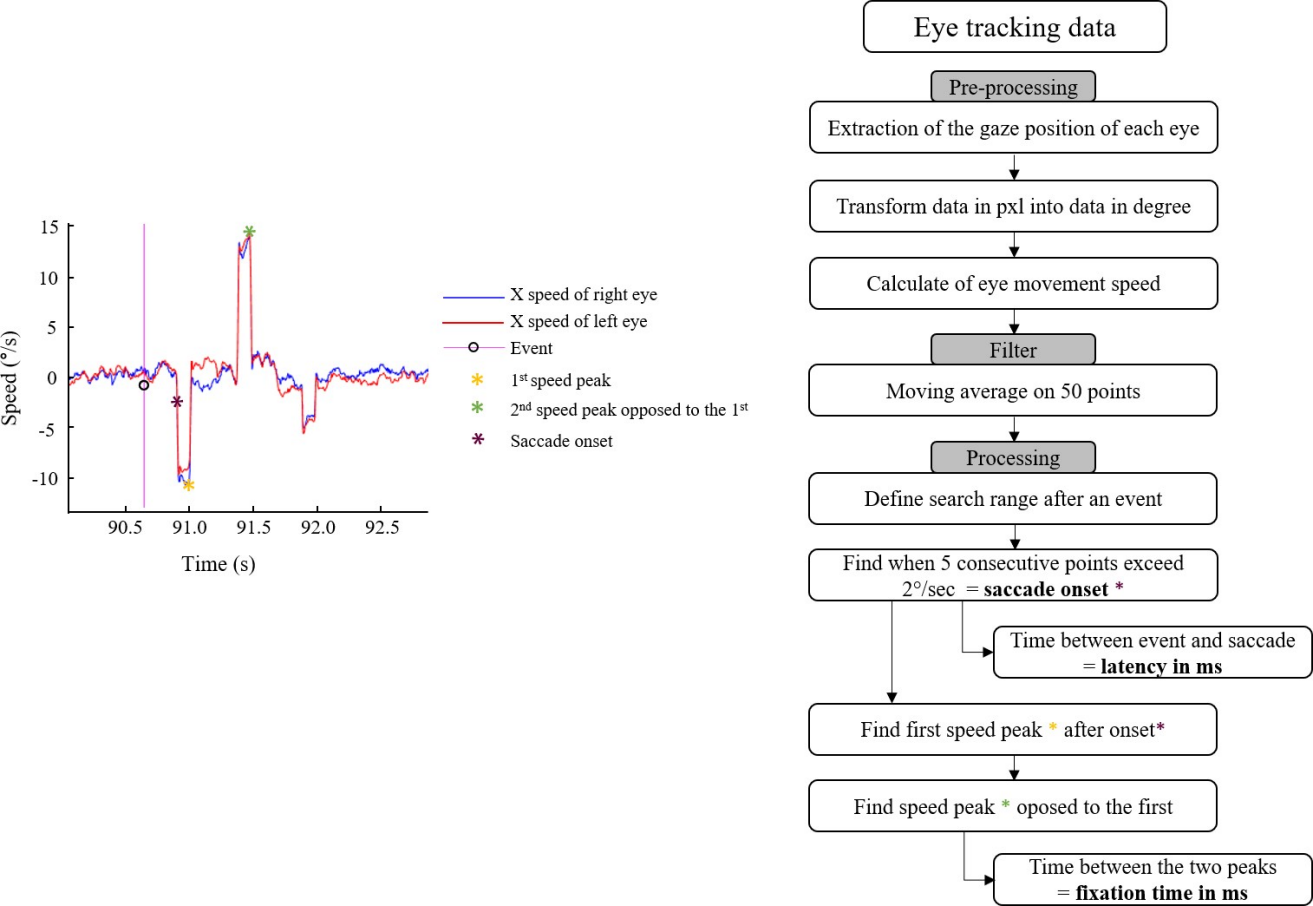
Eye tracking data for a single participant for one event and the analysis pipeline.

Eye tracking data processing is shown in Fig 9. One of the eighteen participants had significant measurement noise in several conditions, which led to his exclusion. Seventeen participants were therefore included in the analyses of *latency* and *event fixation time*.

The participant could indicate a *loss of information* from the target or the monocular element by pressing a trigger at the moment the loss was noted. The total press time in seconds, was recorded for each condition. At the end of each condition, during a break, the participant was asked to orally confirm whether there had been a loss of visual information from the binocular target, the monocular element, or the background (subjective signal loss).

The evaluation of visual comfort provided a score for each of the items assessed.

Performance and visual comfort were determined as a function of background display parameters and the monocular element, which could have four modes: two levels of contrast, and two pulsing profiles (with or without).

### Statistical analyses

Statistical analyses were performed using Statistica (Tibco Software™, Palo Alto, CA, United States). Given the quantitative nature of the data, the fact that the same participants were exposed to all 32 conditions, and the need to compare conditions across multiple factors, a multi-factor repeated measures ANOVA was done. When a *post-hoc* test was run, in all cases the Fisher test was used. When the repeated measures considered only one factor with two modalities paired t-tests were run.

Repeated measures ANOVA and paired *t*-tests were run on the measured variables, and are described below. The conditions of application of these parametric tests have been checked. The level of significance was set to *α* = 0.05 in all tests.

Given that the identification score was 98.8% correct for all participants, *quality of event identification* was not statistically analyzed. Furthermore, the *loss of information* reported via the questionnaire and through trigger pressing, and *visual comfort* were not analyzed, given the small data sample.

#### The effect of the display eye

To evaluate the effect of the display eye, a paired *t*-test was run on the four dependent variables, with the monocular element on the right and left eye for each participant.

#### The effect of interocular conflict on motor fusion

Optometric measurements before and after each session (each comprising 16 conditions) were compared using paired *t*-tests.

#### Target tracking performance

A repeated measures ANOVA of target tracking was run for background characteristics (spatial frequency and contrast), and monocular element characteristics (contrast and pulsing). The factors associated with the event display mode could not be included in the target tracking analysis, because this variable is based on a mean associated with each condition, and not a single occurrence of one condition.

#### Event detection, latency, and fixation

A repeated measures ANOVA was run for *event detection* time, and the two eye tracking measurements (*latency* and *fixation*), with background characteristics and monocular element characteristics, along with the event display mode (monocular or binocular, and small or large eccentricity).

## Results

### The effect of the display eye

Regardless of the binocular background or monocular element characteristics, display eye only had an effect on *target tracking* performance for four participants (see Table 1). In five cases, there was an effect of display eye on *event detection* time (in only one case target tracking performance was a function of the display eye). Finally, in two cases there was an effect of display eye on *fixation* time, but there was no effect of *latency*.

**Table 1:** Distribution of participants as a function of their best eye (RE = right eye; LE = left eye).

Variable	Right eye better		Left eye better		Not significant		N
**Target tracking**	**N = 1**		**N = 3**		**N = 14**		**18**
	**RE**	**LE**	**RE**	**LE**	**RE**	**LE**	
M ± SD	8.15 **±** 0.79	10.06 **±** 1.67	11.15 **±** 1.57	10.31 **±** 1.47	11.08 **±** 2.44	11.04 **±** 2.66	
**Event detection**	**N = 3**		**N = 2**		**N = 13**		**18**
	**RE**	**LE**	**RE**	**LE**	**RE**	**LE**	
M ± SD	825.71 **±** 113.87	900.43 **±** 131.99	894.29 **±** 89.99	835.79 **±** 86.27	802.87 **±** 124.55	810.43 **±** 135.64	
**Latency**	**N = 0**		**N = 0**		**N = 17**		**17**
	**RE**	**LE**	**RE**	**LE**	**RE**	**LE**	
M ± SD					254.14 **±** 21.67	255.05 **±** 21.17	
**Fixation**	**N = 1**		**N = 1**		**N = 15**		**17**
	**RE**	**LE**	**RE**	**LE**	**RE**	**LE**	
M ± SD	286.91 **±** 27.23	319.10 **±** 39.40	406.35 **±** 60.27	329.52 **±** 29.20	322.32 **±** 60.77	330.50 **±** 64.32	

This effect of the display eye was also tested under each binocular background condition (see Table 2). The degradation of the background does not imply a greater difference between the two eyes. For those participants in whom one eye performs better, it is not better regardless of the variable. For one participant, the better eye is even different depending on the variables.

**Table 2:** Paired t-test between the right and the left eye performances for each participant under the four background conditions.

	HSF-100 %				LSF-100 %				LSF-60 %				LSF-20 %			
	Track.	Detect.	Lat.	Fix.	Track.	Detect.	Lat.	Fix.	Track.	Detect.	Lat.	Fix.	Track.	Detect.	Lat.	Fix.
**S1**	NS	NS	NS	NS	NS	NS	NS	NS	NS	NS	NS	NS	NS	**RE**	NS	NS
**S4**	**LE**	NS	NS	NS	NS	NS	NS	NS	NS	NS	NS	NS	NS	NS	NS	NS
**S6**	NS	NS	NS	NS	NS	NS	**RE**	NS	NS	NS	NS	NS	NS	NS	NS	NS
**S7**	NS	NS	NS	**RE**	NS	NS	NS	NS	NS	NS	NS	NS	NS	NS	**RE**	NS
**S8**	NS	NS	NS	NS	NS	**RE**	NS	NS	NS	NS	NS	NS	NS	NS	NS	NS
**S9**	**LE**	NS	⍉	⍉	**LE**	NS	⍉	⍉	NS	NS	⍉	⍉	NS	NS	⍉	⍉
**S10**	**RE**	**RE**	NS	NS	NS	NS	NS	NS	NS	NS	NS	NS	NS	NS	NS	NS
**S11**	NS	NS	NS	**RE**	NS	NS	NS	NS	NS	NS	NS	NS	NS	NS	NS	NS
**S12**	NS	**RE**	NS	NS	NS	NS	NS	NS	NS	NS	NS	NS	NS	NS	NS	NS
**S13**	NS	NS	NS	NS	NS	NS	NS	NS	NS	**RE**	**RE**	NS	NS	NS	NS	NS
**S14**	NS	NS	NS	NS	NS	**LE**	NS	NS	NS	NS	NS	NS	**LE**	NS	NS	NS
**S16**	NS	NS	NS	NS	**RE**	NS	NS	NS	NS	NS	NS	NS	NS	NS	NS	NS
**S17**	NS	NS	NS	NS	NS	**RE**	NS	NS	NS	NS	NS	NS	NS	NS	NS	NS
**S18**	NS	NS	NS	NS	**RE**	NS	NS	**LE**	NS	**LE**	NS	**LE**	NS	NS	NS	NS
**TOTAL**	N = 3	N = 2	N = 0	N = 2	N = 3	N = 3	N = 1	N = 1	N = 0	N = 2	N = 1	N = 1	N = 1	N = 1	N = 1	N = 0
**Better eye** M ± SD	10.53 ± 3.45	882.36 ± 288.20		260.49 ± 17.41	8.80 ± 0.85	760.41 ± 83.82	221.59	316.56		829.57 ± 115.23	253.93	284.72	10.32	817.96	219.57	
**Worse eye** M ± SD	11.68 ± 3.53	1000.97 ± 393.41		321.38 ± 14.94	10.74 ± 0.47	821.81 ± 88.20	269.58	419.97		959.18 ± 196.15	337.57	396.52	11.61	878.69	249.37	

### The effect of interocular conflict on motor fusion

Fusion capabilities were assessed by optometric tests before and after each session. No significant differences were found, indicating that our experiment did not alter the oculomotor capacities of participants (see Table 3).

**Table 3:** Results and statistical analyses before and after each of the three optometric tests in the two sessions.

	Session 1			Session 2		
	Pre-test	Post-test	*p-value*	Pre-test	Post-test	*p-value*
	M ± SD	M ± SD		M ± SD	M ± SD	
**Phoria**	0.08 ± 1.33	0.33 ± 1.36	0.58	0.33 ± 1.52	0.42 ± 0.42	0.88
**Disparity**	-0.14 ± 0.45	-0.19 ± 0.89	0.82	-0.14 ± 0.76	-0.11 ± -0.11	0.91
**Fusional reserves**						
Blur BI	5.44 ± 1.50	5.89 ± 2.32	0.50	5.89 ± 1.20	6.11 ± 2.22	0.75
Break BI	6.67 ± 1.94	6.89 ± 2.30	0.76	7.11 ± 1.97	7.33 ± 1.94	0.74
Recovery BI	4.67 ± 1.94	4.78 ± 2.07	0.87	5.11 ± 1.97	5.44 ± 2.15	0.63
Blur BO	10.56 ± 4.59	10.67 ± 3.82	0.94	10.33 ± 4.19	11.33 ± 3.69	0.45
Break BO	22.89 ± 8.52	23.28 ± 9.94	0.90	23.83 ± 9.93	24.39 ± 10.96	0.87
Recovery BO	17.83 ± 5.95	18.56 ± 9.08	0.78	17.94 ± 7.08	19.33 ± 9.63	0.63
**Forced vergence**						
3Δ BI	0.14 ± 0.34	0.39 ± 0.88	0.27	0.25 ± 0.55	0.25 ± 0.65	1
6Δ BI	0.39 ± 0.50	0.75 ± 0.58	0.05	0.50 ± 0.57	0.69 ± 0.79	0.40
10Δ BI	1.64 ± 0.95	1.64 ± 0.95	0.80	1.69 ± 0.94	1.62 ± 1.11	0.69
3Δ BO	-0.58 ± 1.02	-0.47 ± 0.98	0.74	-0.25 ± 0.58	-0.47 ± 1.09	0.45
6Δ BO	-0.77 ± 0.89	-0.94 ± 0.97	0.74	-0.64 ± 1.14	-0.74 ± 1.16	0.42
10Δ BO	-1.19 ± 2.14	-1.69 ± 1.61	0.43	-0.91 ± 1.05	-1.03 ± 1.04	0.86

BO = Base-out; BI = Base-in.

### The effect of stimuli characteristics

#### Target tracking performance

**Fig 10:**
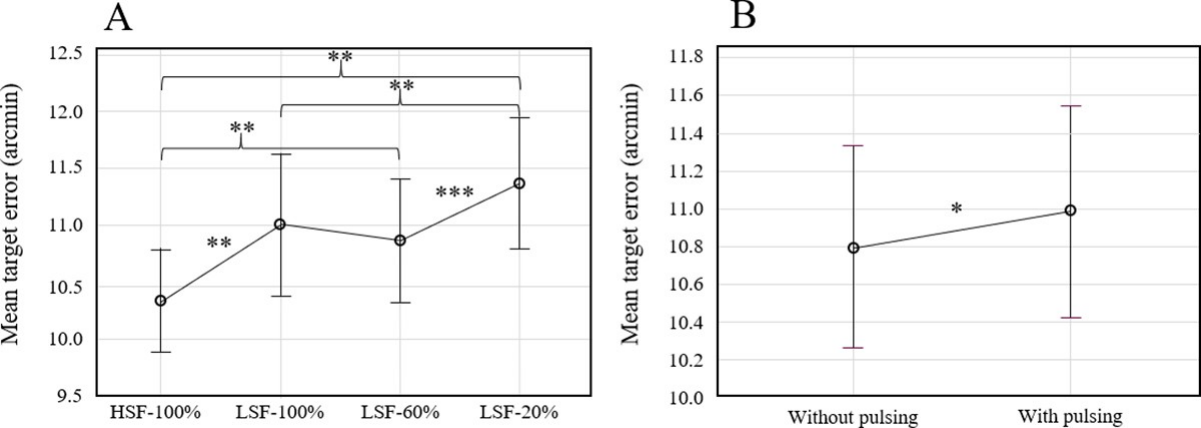
From left to right, respectively mean target error considering binocular background characteristics (A) and with or without pulsing (B). Error bars represent standard error, and main effects are indicated as follows: * *p* < 0.05, ** *p* < 0.01, *** *p* < 0.001.

The repeated measures ANOVA of target tracking, considering the binocular background characteristics and monocular element configurations, revealed an effect of background characteristics, F(2.1,35.79) = 14.72, *p* < 0.001 (see Fig 10A). A *Post-hoc* analysis revealed a main effect of spatial frequency. Performance was significantly better (lower mean error between the center of the monocular element and the center of the binocular target) in the HSF-100% condition (M = 10.33arcmin; SD = 1.95 arcmin) compared to the LSF-100% condition (M = 11.01 arcmin; SD = 2.74 arcmin). A contrast effect was observed at low spatial frequencies. Target tracking performance was significantly poorer in the LSF-20% condition (M = 11.37 arcmin; SD = 2.57 arcmin) compared to both LSF-60% (M = 10.86 arcmin; SD = 2.34’) and 100% (M = 11.01 arcmin; SD = 2.74 arcmin) conditions. On the other hand, there was no significant difference between LSF-100% and LSF-60% conditions.

A main effect of pulsing was also identified, F(1,17) = 4.53; *p* = 0.048 (see Fig 10). Specifically, performance was poorer in the pulsing condition (M = 10.99 arcmin; SD = 2.50 arcmin) compared to the condition without pulsing (M = 10.80 arcmin; SD = 2.38 arcmin). The contrast of the monocular element had no effect on tracking performance.

#### Quality of event identification

The number of identification errors recorded by all participants in all conditions was 110 out of a total of 9216 events displayed. As the correct identification rate was 98.81%, no statistical analysis was performed to assess the distribution of errors according to the characteristics of the stimuli.

#### Event detection, latency, and fixation

**Fig 11:**
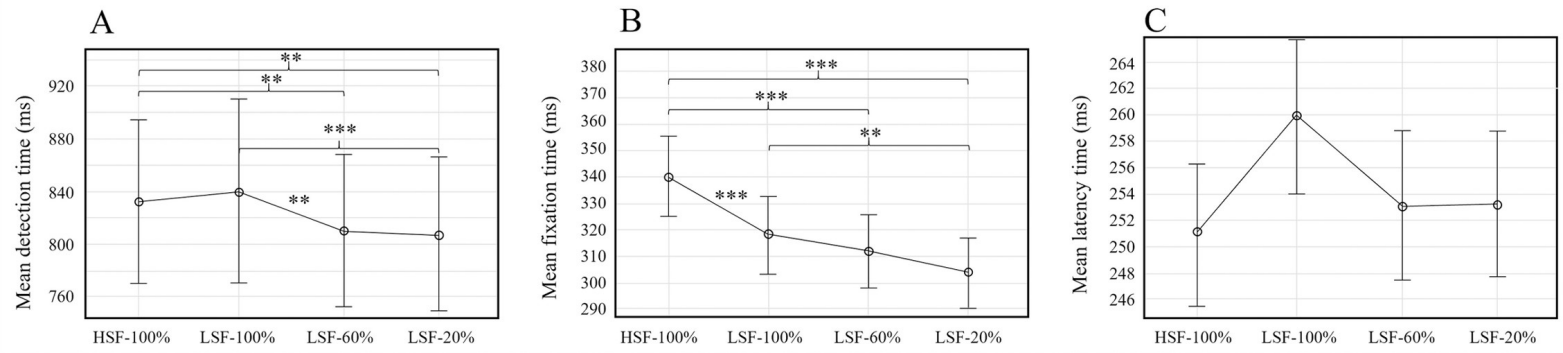
From left to right respectively, mean times for *detection* (A), *fixation* (B) and *latency* (C) as a function of the characteristics of the binocular background. Error bars represent standard error and main effects are indicated as follows: * *p* < 0.05, ** *p* < 0.01 and *** *p* < 0.001.

*The effect of the binocular background configuration*. Analyses of *event detection time* as a function of background characteristics found a significant effect, F(2.1,35.67) = 7.83; *p* < 0.01 (see Fig 11). The *post-hoc* analysis revealed an effect of contrast, but not spatial frequency, as no effect was found between HSF-100% and LSF-100% conditions. However, it increased significantly in the LSF-100% condition (M = 839.12 ms; SD = 141.69 ms) compared to the LSF-60% condition (M = 810.24 ms; SD = 118.03 ms) and the LSF-20% condition (M = 805.58 ms; SD = 124.69 ms).

No effect of binocular background was found for *latency*, F(2.59,41.38) = 1.86; *p* = 0.0.16 (see Fig 11), but an effect was found for *fixation time*, F(2.25,36.06) = 16.51, *p* < 0.001 (see Fig 11). The *post-hoc* analysis found an effect of spatial frequency; *fixation time* was significantly shorter for the LSF-100% condition (M = 315.74 ms; SD = 63.09 ms) than for the HSF-100% condition (M = 340.20 ms; SD = 61.91 ms). A contrast effect was also observed. *Fixation time* was significantly shorter for the LSF-20% (M = 303.12 ms; SD = 54.23 ms) compared to the LSF-100% condition (M = 315.74 ms; SD = 63.09 ms). On the other hand, no significant difference was found between LSF-100% and LSF-60% conditions (M _=_ 312.18 ms; SD = 57.00 ms), or between LSF-60% and LSF-20% conditions.

*The effect of monocular element characteristics and event display mode*. The impact of the monocular element characteristics (contrast and pulsing) and the event display mode (monocular or binocular, and small or large eccentricity) on *event detection*, *latency* and *event fixation* time was studied for each binocular background configuration:

- high spatial frequency and 100% contrast (HSF-100%);
- low spatial frequency and 100% contrast (LSF-100%);
- low spatial frequency and 60% contrast (LSF-60%);
- low spatial frequency and 20% contrast (LSF-20%).

**Fig 12:**
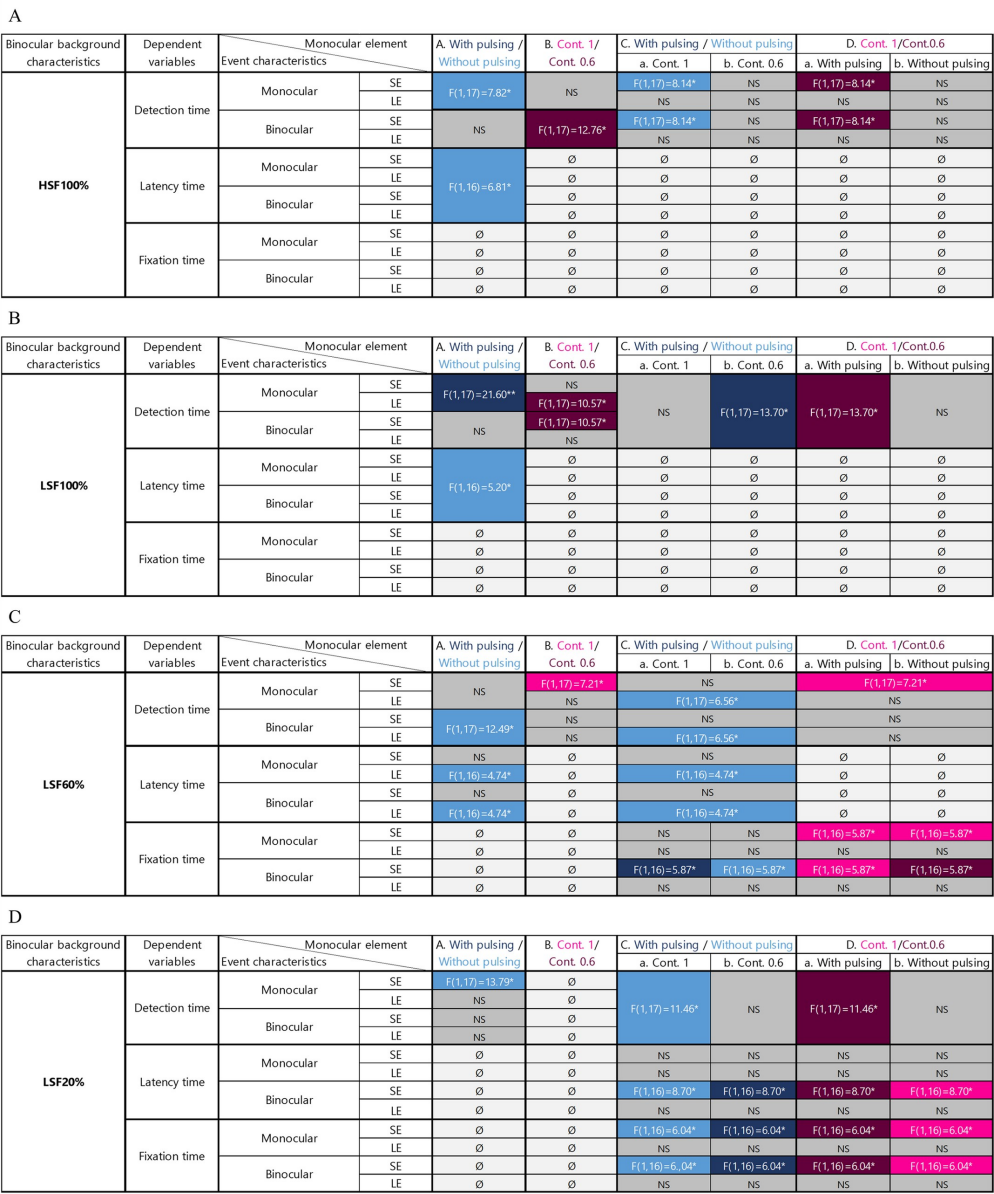
Repeated measures ANOVA of *event detection*, *latency* and *fixation times* as a function of the characteristics of the monocular element and the event display mode in the HSF-100% (A), LSF-100% (B), LSF-60% (C) and LSF-20% (D) conditions. In these tables, event display parameters are presented in rows and the characteristics of the monocular element are shown in columns. SE = small eccentricity (0.81°); LE = large eccentricity (2.00°). A non-significant interaction is reported as NS. Where no interaction was found, the symbol Ø is used. In all cases, Column A evaluates the interaction between event display characteristics and pulsing. Where a *F* statistic is shown, the *post-hoc* test revealed either a beneficial effect of the pulsation (dark blue box) or a detrimental effect (light blue box). Column B evaluates the interaction between event display and monocular element contrast. Where a *F* statistic is shown, a pink box indicates a beneficial effect when the maximum contrast of the monocular element was set to 1, and a dark purple box indicates a beneficial effect when it was set to 0.6. Columns C and D show three-way interactions between event display, pulsing and monocular element contrast. Column Ca shows differences in performance between the condition in which the monocular element is pulsed and the condition without pulsing at maximum contrast (1). Column Cb shows the same effect of pulsing at 0.6 contrast. In both cases, a dark blue box indicates a beneficial effect of pulsing compared to the same condition without pulsing. Column Da indicates differences in performance between the condition with the monocular element at maximum contrast set to 1 or 0.6 with pulsing. Column Db shows the results for the same condition, but without pulsing. Here, a pink box indicates a beneficial effect of contrast 1 compared to 0.6, and a dark purple box indicates a beneficial effect of 0.6 contrast.

The results of the repeated measures ANOVA for factors associated with the characteristics of the monocular element and the event display mode are summarized in Fig 12 according to each binocular background condition.

Fig 12A shows that, for the HSF-100% condition, overall performance is better without pulsing (column A: *detection time* and *latency*) than with pulsing. If the contrast of the element is set to 0.6, there is no difference with or without pulsing (column Cb and Da: *detection time*). If, however, contrast is set to 1, the monocular element should not be pulsed (column Ca).

In LSF-100% condition (see Fig 12B), performance is better at maximum contrast 0.6 (column B: *event detection time*) than at 1. If the contrast of the element is set to 0.6, the monocular element should be pulsed (columns Cb and Da: *event detection time*).

For the LSF-60% condition (see Fig 12C), performance is better with the monocular element set at maximum contrast 1 (column B: *event detection time* and column D: *event detection time* and *fixation time*), with pulsing (columns Ca: *fixation time*)–or with the monocular element set at maximum contrast 0.6 (columns B and D: *latency*), without pulsing (column Cb: *fixation time* and *latency time*).

In order to identify if one of the latter two options was preferable, a *post-hoc* analysis was run to compare them for each variable (see Table 4). This found no significant difference between results with maximal contrast set to 1 with pulsing, and with maximal contrast set to 0.6 without pulsing except for *latency time*.

**Table 4:** Comparison between two options in LSF-60%.

	Contrast 1 with pulsing	Contrast 0.6 without pulsing	*p-value*
***Detection time***	808.34 ± 134.40	802.13 ± 140.60	0.64
***Latency time***	258.78 ± 50.70	242.90 ± 36.44	0.004
***Fixation time***	305.97 ± 82.94	322.18 ± 72.75	0.13

Finally, for the LSF-20% condition (see Fig 12D), performance is better with the monocular element set at maximum contrast 1 without pulsing (Column Ca: *event detection time*, *latency* and *fixation time* and column Db: *latency* and *fixation time*) or with the monocular element set at maximum contrast 0.6 with pulsing (column Cb: *latency* and *fixation time* and column Da: *event detection time*, *latency* and *fixation time*).

In order to identify, if one of the two options must be favored, a post-hoc analysis compared them for each variable (see Table 5). There is no significant difference between results in contrast 1 without pulsing and contrast 0.6 with pulsing for the LSF-20% conditions.

**Table 5:** Comparison between two options in LSF-20%.

	Contrast 1 without pulsing	Contrast 0,6 with pulsing	*p-value*
***Detection time***	803.26 ± 121.19	785.01 ± 162.82	0.13
***Latency time***	250.38 ± 26.71	257.71 ± 43.65	0.46
***Fixation time***	300.50 ± 75.83	309.17 ± 68.96	0.52

Regardless of the binocular background condition, *detection*, *latency* and *fixation* times were shorter for small eccentricity events than large eccentricities. Finally, these times were shorter in 67% of conditions when the event was binocular rather than monocular.

#### Loss of information

Only five of the 18 participants reported at least one loss of information by pressing the trigger and then confirmed it orally; this number was insufficient for analysis. The cumulative time to report loss of information was 0.16% of the total time allocated to all conditions for the 18 participants.

#### Visual comfort

The questionnaire did not reveal any significant discomfort, and levels never exceeded 2/5 regardless of the binocular background condition (see Table 6). The score for each item was measured and calculated according to the participants’ pre-session status and then averaged.

**Table 6:** Mean visual comfort scores as a function of the binocular background conditions.

Visual comfort	HSF-100 %	LSF-100 %	LSF-60 %	LSF-20 %
**Visual fatigue**	0.59 ± 0.91	0.68 ± 1.01	0.58 ± 0.92	0.72 ± 1.06
**Headaches**	0.19 ± 0.46	0.18 ± 0.42	0.15 ± 0.39	0.15 ± 0.41
**Double vision**	0.05 ± 0.25	0.04 ± 0.20	0.07 ± 0.31	0.11 ± 0.39
**Blurred vision**	0.25 ± 0.60	0.45 ± 0.88	0.45 ± 0.89	0.45 ± 0.96
**Eye tightness**	0.45 ± 0.86	0.47 ± 0.79	0.41 ± 0.77	0.36 ± 0.70

N = 18, 0 = no discomfort, 5 = very intense discomfort.

## Discussion

The aim of this study was to evaluate the impact of different display conditions on performance and visual comfort in the presence of interocular conflict, such as that found in a monocular see-through near-eye display.

We evaluated the effect of the characteristics of the binocular background, the monocular element, and the event display mode on performance and visual comfort and, consequently, the impact on the interocular conflict. Using an experimental projector-based device, we measured the following variables: performance in tracking a binocular target; the detection and identification of monocular and binocular events; latency and fixation time and visual comfort. All of these variables were measured as a function of several levels of spatial frequency, contrast of the binocular background, with a monocular element with variable contrast that may (or may not) stimulate exogenous attention. These configurations were also evaluated as a function of the eye on which the monocular element was displayed.

### The effect of display eye

As there is no consensus in the literature regarding the impact of ocular dominance during interocular conflict, we adopted the above protocol to evaluate differences in tracking performance, detection and identification when the monocular image was displayed on the right eye, and when it was displayed on the left eye. Only four participants had better tracking performance with one eye than the other (see Table 1). Only one of these four also had a difference in detection time when the monocular element was displayed on one eye (it turned out that this was not the dominant eye for this participant). Five participants had shorter detection times with the monocular element on one eye compared to the other, and for two others fixation time differed (see Table 1).

As Brown et al. [7] have suggested, we compared the impact of the display eye according to binocular background conditions. Our results suggest that the impact of the display eye on performance does not depend on the binocular background conditions.

Our results were observed with a stimulus that matched the size of the chosen monocular element (0.7°), and it will be necessary to verify our findings with stimuli such as flight symbology, which occupies a larger monocular field and increases interocular conflict.

### The effect of interocular conflict

Very few (five) participants reported loss of information, and it represented a very small amount of time compared to the total exposure time (0.16%). The stimuli and tasks chosen for this study seem to have limited the suppression phenomenon, as disturbances attributed to binocular rivalry were limited to performance degradation that was a function of the characteristics of the binocular background and the monocular display. This low rate information loss may be explained by the coherence between the monocular element (a ring) and the binocular target (a circle), which seems to limit rivalry [33].

It should also be noted that the selected stimuli and tasks did not disrupt motor fusion. This may be directly related to the small amount of information loss, which suggests that sensory fusion was able to take place, supporting motor fusion [34]. This is consistent with the low double vision score reported by participants (see Table 6). As vision was neither doubled nor suppressed, it appears to have been fused.

The number of misidentifications across all participants in all conditions was very low (1.2%), and exposure only led to limited complaints.

The low proportion of information loss, the absence of motor fusion modification, the low number of misidentifications and the high level of comfort suggest that the selected stimuli and tasks were not very demanding, and did not cause difficulties to participants. Although not strictly speaking ecological, our study nevertheless simulated an interocular conflict that can be replicated. Our results are therefore encouraging in terms of user acceptance of a system that creates this type of interocular conflict. However, the failure to identify any symptoms, which, according to some authors are characteristic of monocular augmented reality displays [3, 4, 6] could suggest that certain of our methodological choices made it difficult to account for all the constraints associated with these displays, as we found that binocular rivalry was more limited than we would have predicted.

As our aim was to evaluate the interocular conflict effect on performance, we chose to limit the participant’s head movements. It would be interesting to test our results in a more ecological study of monocular see-through HMD considering the slaving of the virtual image with the head movement.

### The effect of binocular background characteristics

#### Spatial frequency impacts tracking performance and predominance

We evaluated the effect of the binocular background spatial frequency on performance. In the second experiment reported by Hershberger and Guerin [6], target tracking performance is measured as a function of two types of see-through HMD (good or poor) on an identical binocular background. Their results show that whatever the difference in spatial frequency between the monocular image and the background, tracking performance is not impaired. Surprisingly, our results do not support their findings, as we find better tracking performance with a high spatial frequency background compared to a low spatial frequency (see Fig 10A). However, the first experiment reported by Hershberger and Guerin [6] highlights the effect of a difference in spatial frequency. It shows that the greater the difference in spatial frequency between conflicting elements, the more the element with the higher frequency predominates. Thus, low background spatial frequencies (compared to the high frequencies of the monocular element) are likely to have had the effect of making the monocular element too predominant relative to the background and target, making tracking more difficult (see Fig 10A). Our results for fixation time also support this argument. Fixation time was significantly shorter for low spatial frequency background conditions, compared to high frequency conditions (see Fig 11B). The high spatial frequency event dominated the low spatial frequency background, reducing conflict, and therefore shortening fixation times.

#### Contrast impacts tracking performance

Performance and comfort were also evaluated with different binocular background contrasts. While previous studies have only varied the contrast of the monocular element [6, 7, 35], our study compared the impact of contrast variation of the binocular background on interocular conflict. Our findings show that, regardless of the contrast of the monocular element, tracking performance was better in the LFS-100% condition than in LFS-20% condition (see Fig 10A).

In our experiments, the binocular target is so large (2.1°) that the monocular element is not usually superimposed on the background but remains limited to the target. This target has a mean luminance that does not vary with background conditions (13 cd/m^2^). As our results show that performance is not dependent on the monocular element contrast, and the target has a fixed luminance, it is not the difference in contrast between these two elements that degrades performance–as some authors have suggested [21, 22, 36]. Therefore, in our experiments, poorer target tracking performance as a function of background contrast appears to be due to the difference in contrast between the binocular target and the binocular background. In practice, as one would expect, it may be more difficult to track a target on a low contrast background than one with high contrast.

#### Contrast impacts event detection time and predominance

In our work, the contrast of the event to be detected does not change under any condition (it is fixed at 100%), and event detection time was shorter when background was in LSF-60% and 20% (see Fig 11A). It should be noted that in these conditions there is no contrast difference between the event and the binocular background. This result is in agreement with studies that evaluate dichoptic images in which the higher contrast image dominates perception [21, 36]. It is also in agreement with the results of Winterbottom et al. [35], who found better high contrast target detection performance on a low contrast background.

#### Contrast impacts event fixation time

The impact of contrast on fixation time has not been tested in other studies that use a monocular image superimposed on a binocular background. Our results suggest that the higher the contrast of the stimuli, the shorter the fixation time required to identify it. These results are in agreement with results obtained with binocular images [37].

### The effect of display characteristics

#### Monocular/binocular display impacts event detection time

We also studied the impact of event display on performance. Winterbottom et al. [35] used a monocular image superimposed on a binocular background. Their study found that event detection and identification performance improved when the event appeared on the binocular background compared to the monocular image. Our study evaluated this performance under four binocular background conditions, with events presented either monocularly or binocularly. Regardless of the characteristics of the binocular background, detection times of the event was longer when it was monocular than when it was binocular. Our results are in agreement, and complement those of Winterbottom et al. [35], as the latter study only tested the impact on performance under optimal flying conditions.

#### Eccentricity impacts event detection performance

As in natural vision conditions [38] and as one would expect, under interocular conflict, detection performance is better for an event at small eccentricity than at large eccentricity.

While our results demonstrate differences in detection times for monocular and binocular events, and between events displayed at small and large eccentricity, these parameters are not under the control of the observer, and cannot be anticipated. Consequently, our results were examined to determine which display configuration (in terms of contrast and pulsing) is preferable for each environmental configuration.

#### Contrast and pulsing of monocular element impact tracking performance and event detection performance

Overall, it seems that the best way to limit the impact of interocular conflict on perceptual performance is to display the monocular element with pulsing at intermediate contrast. The only exception is the LSF-60% condition, where either pulsing at maximum contrast, or without pulsing at intermediate contrast is better. The contribution of the pulsing must be nuanced since it seems to degrade tracking performance regardless of maximum contrast.

While the study of endogenous attention seems to be of little interest [28], exogenous attention (stimulated by pulsing) seems to limit the impact of interocular conflict, if the target is clearly specified with respect to the contrast level. It should be noted that in our study exogenous attention may have been stimulated by the presence of events, as 16 were displayed during each two-minute configuration. Thus, it is possible that rivalry was reduced by the appearance of the event, rather than the presence of the pulse; this would explain why few participants reported subjective monocular element loss. Future work should examine the role played by the pulsing, by limiting the stimulation of exogenous attention by other, non-ecological phenomena.

Intermediate contrast could be used in all conditions, by modulating it with the stimulation of exogenous attention. The overall superiority of this level of contrast could be linked to the fact that in maximum contrast, without pulsing, the monocular element predominates [6] to such an extent that it prevents the correct detection and identification of surrounding events. Regarding pulsing in maximal contrast, the fact that it oscillates between 1 and 0 leads to too much information being lost from the monocular element, creating stress, and degrading the capacity of the participant to detect the event. It would be interesting to test a narrower range of contrast, and avoid a total loss of information, in order to check if the poor results obtained in maximum contrast can be overcome.

From a qualitative point of view, our findings using a dynamic background are broadly similar to those reported by Hershberger and Guerin [6] who used static backgrounds.

## Conclusion

Our results show that the interocular conflict generated when a monocular image is superimposed on a binocular background affects performance, as a function of the characteristics of the binocular background and the monocular image. Our findings suggest that it is possible to determine a display configuration that limits this conflict. It seems to be important that the virtual image displayed in a see-through HMD can be set up or adapted to environmental conditions. Our results regarding the monocular element with pulsing imply that the use of pulsed symbology in a monocular see-through HMD is a promising area for further investigation, and could limit the effects of interocular conflict. However, our preliminary results need to be confirmed in a more ecological environment to determine precisely when, and how, to adjust the display configuration.

## Supporting information

S1 Data Average tracking performance per participant (N = 18) based on the 32 display conditions tested. (XLSX)

S2 Data Average detection time per participant (N = 18) across the 32 display conditions tested and according to event display. (XLSX)

S3 Data Average fixation time per participant (N = 17) across the 32 display conditions tested and according to event display. (XLSX)

S4 Data Average latency time per participant (N = 17) according to the 32 display conditions tested and according to the event display. (XLSX)
